# Independent living transitions for young people with cerebral palsy in Australia: aligning policy and practice with family realities

**DOI:** 10.3389/fpubh.2026.1755553

**Published:** 2026-02-19

**Authors:** L. Hickey, H. T. D. Nguyen, L. Harms, E. Culnane, V. Saunders, C. Imms, M. Ball, D. Reddihough

**Affiliations:** 1 Department of Social Work, The University of Melbourne, Melbourne, VIC, Australia; 2 CP-Achieve NHMRC Centre of Research Excellence, Murdoch Children’s Research Institute, Melbourne, VIC, Australia; 3 Neurodevelopment and Disability, The Royal Children’s Hospital, Melbourne, VIC, Australia; 4 Transition Support Service, The Royal Children’s Hospital, Melbourne, VIC, Australia; 5 Young Adult Complex Disability Service, St. Vincent’s Hospital, Melbourne, VIC, Australia; 6 Department of Paediatrics, The University of Melbourne, Melbourne, VIC, Australia

**Keywords:** cerebral palsy, family, family adaptation, resiliency, transition to independent living

## Abstract

The transition to independent living is a key marker of adulthood for many adolescents and young adults (AYAs) with disability. How families adapt to this highly complex process is an important area for examination, to inform policy and practice that effectively support family life. This study aimed to understand family adaptation processes during the independent living transitions for families of AYAs with cerebral palsy. Semi-structured interviews were conducted with 13 participants who identified as parents of an AYA with cerebral palsy. Data were analysed deductively using the Resiliency Model of Family Stress, Adjustment, and Adaptation highlighting the vulnerability and resiliency factors that shape family adaptation. Vulnerability factors related to a pile-up of demands in family life, particularly the transitions out of the school and paediatric health system that occurred around the same time as the transition to independent living. Resiliency factors included family appraisal processes, family resources, problem-solving capacities, coping strategies, and patterns of family functioning. Participants highlighted the systemic challenges that impacted family adaptation, namely rigid policies, unfamiliar and complex bureaucratic processes, market-driven services, and disability workforce issues. Findings highlight the importance of a family systems perspective in both policy and practice. Policy needs to respond to the needs of both the AYA and their ageing parents, as well as changes in family demands and resources over time. The practice implications are that family involvement, strengths, and expertise are valued and promoted, to support the independent living transition specifically, and family life more broadly.

## 1 Introduction

Moving into independent living is one of the key markers of adult life. It is a complex and multifaceted process for all adolescents and young adults (AYAs) (1, 2). Like their peers, AYAs with cerebral palsy (CP) want to participate in life situations that define adulthood, including the transition to independent living, despite the barriers posed by their condition and imposed by structural contexts (3–5). An understanding of this transition and the barriers, strengths experienced by AYAs with CP and their families is required to inform policy and practice to effectively support families during this crucial life stage (6).

CP presents unique challenges for young people. Cerebral palsy (CP) is an *“early-onset lifelong neurodevelopmental condition characterized by limitations in activity due to impaired development of movement and posture, manifesting as spasticity, dystonia, choreoathetosis, and/or ataxia”* (7). It may be accompanied by epilepsy, musculoskeletal problems, and non-progressive disturbances of sensation, perception, cognition, communication, and behaviour (8).

Previous research in high resource countries indicates lower rates of independent living amongst AYAs with CP when compared with peers of a similar age (4, 9–13). Findings point to some of the key barriers and challenges when transitioning to independent living, or transitioning to adulthood more broadly: unmet information needs about support and housing options; and the lack of holistic, family-centred support upon leaving school and paediatric health care systems; reliance on parents and siblings for assistance with activities of daily living; confidence about the quality of care provided by services and facilities; concerns about the health and wellbeing of the AYA during the transfer of care; lack of discussion and information about sexuality and relationships; navigating complex systems and bureaucratic requirements; timely access to funding and equipment; and negative attitudes in the community (1–6, 11, 14, 15).

The barriers and challenges highlighted in the research reflect systemic and policy issues. In Australia, AYAs with CP are likely to be registered with the National Disability Insurance Scheme (NDIS), which provides funding to people with disability to access disability services and supports (16). The recent NDIS Review (17) recognised significant, ongoing challenges with the disability system. There is a gap in housing and living supports, with a lack of information and advice for people with disability to make informed choices about their housing and living situation; a lack of support to prepare for housing and living solutions early; limited opportunities and funding to trial different solutions; and a lack of Specialist Disability Accommodation (SDAs) which meets people’s needs and enables them to maintain their informal support network (17). Furthermore, the Review found that the quality of supports varies greatly across the market-driven service providers, and workforce training and retention issues impact people’s access to quality supports. The NDIS is an “incredibly complex and confusing” system, and its complex interface with mainstream systems (e.g., school education, health) makes navigating multiple systems even more difficult for people with disability (17). The Review recommended the implementation of Housing and Living Navigators who support people with disability and their families to articulate their goals, explore options, and secure suitable funding for housing and living. This need for transition services in young adulthood has been highlighted in existing research, to ensure that AYAs and families are supported in navigating structural considerations and complex systems (1, 6, 18).

Notwithstanding these systemic and policy barriers, research importantly demonstrates some of the key enablers for AYAs with CP specifically, and AYAs with disability and health complexities more broadly, to successfully transition to adulthood. Family context and the family system are particularly important, with family involvement and interdependence shaping different aspects and outcomes of the transition (19). Family members have expectations and provide opportunities for AYAs to demonstrate autonomy, participate in adult activities, take risks, and make their own decisions (20, 21). To adapt to transitions, families undertake significant amounts of work: advocating for the AYA, working creatively with limited resources, making career sacrifices, altering family roles, and persisting through hardship (15). They play a key role in providing daily living support, but also practical, emotional, and advocacy support to find solutions for independent living challenges, before, during, and after the transition (3, 19, 21, 22). They also assist AYAs with decision-making, particularly with financial decisions (19). In fact, family members often make up for the shortfalls arising from systemic issues (6); for example, providing care to the AYA in the family home while waitlisted for housing (23). Furthermore, family members experience emotional challenges associated with prolonged periods of caregiving, uncertainty around the AYA’s lifespan and future, or grief about ‘letting go’ or having someone else taking over the AYA’s care (15, 19, 24). The AYA’s readiness, preparedness, and confidence about independence is linked to their own sense of assurance and ability to ‘let go’ and support the transition (6). Family members find different ways to cope with the stresses of everyday life and engage in meaning-making and reframing processes to promote a positive outlook and hope for the future (6, 15, 19, 24).

Evidence shows that the AYA’s transition to adulthood affects the whole family, and the AYA’s transition is shaped by family adaptation processes and their interface with structural contexts. Therefore, it is important that family members’ perspectives are examined, and family processes promoted during this transition. However, there is little research that explores family processes and adaptation relating to independent living transitions for AYA with CP (6, 25). Policy has recognised the important role of family and carers, as seen as in policy documents such as the NDIS Review (16) and the National Carer Strategy 2024–2034 (26); however, a family systems perspective is lacking. Such policy positions family and carers as supporters of the individual with disability, and not as individuals with needs in their own right (16, 26). While a focus on supporting individual needs, personal agency, and individual decision-making is important, it may overlook how adaptation occurs and is influenced within a family system. For example, the Housing and Living navigator role is recommended to support NDIS participants with their choice and control over housing and living support; family processes such as advocacy, decision-making, and meaning making remain largely invisible.

**Figure 1:**
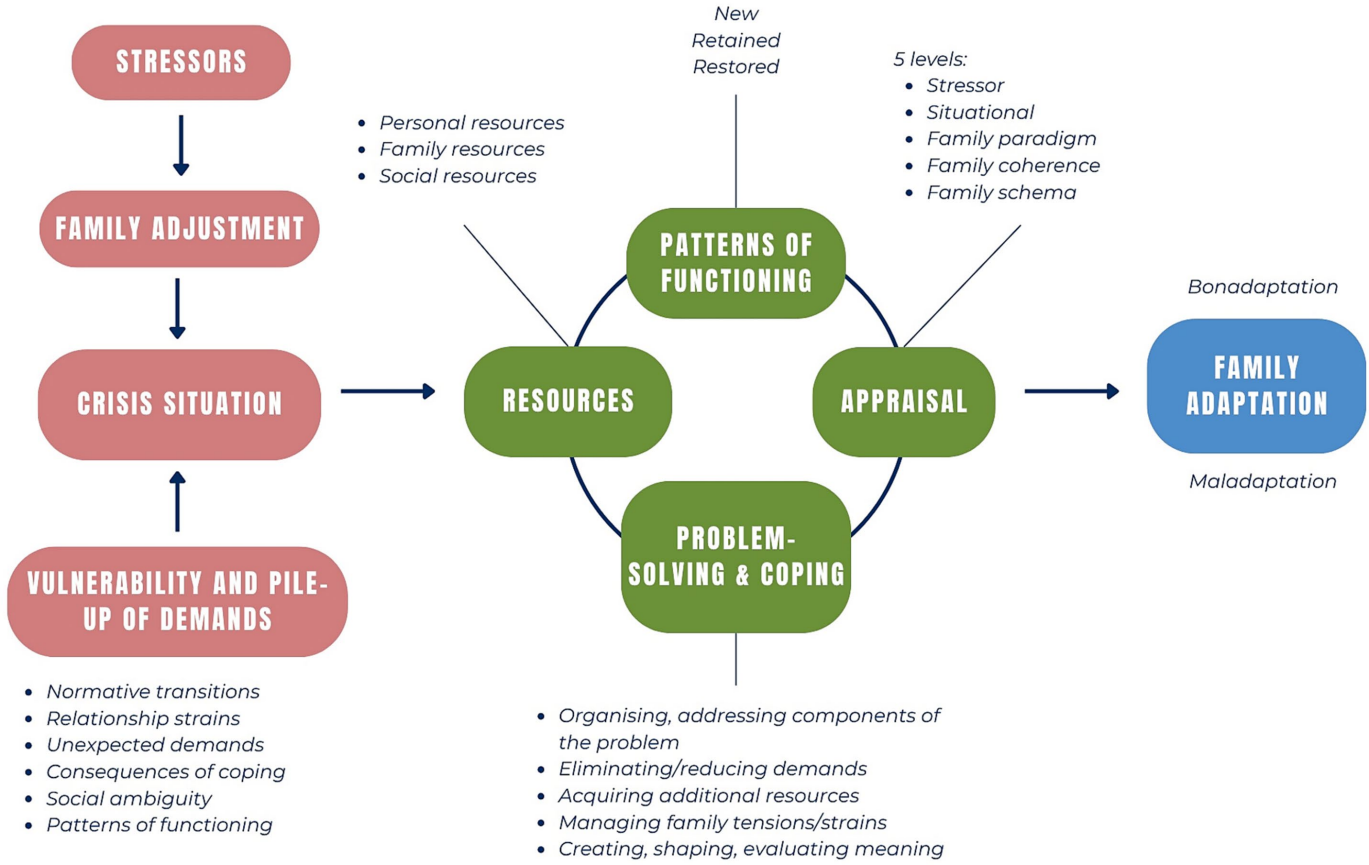
The resiliency model of family stress, adjustment, and adaptation. Adapted from McCubbin and McCubbin (27), McCubbin et al. (31) and Spina et al. (32).

The Resiliency Model of Family Stress, Adjustment and Adaptation (27) provides a useful theoretical basis for understanding family processes and adaptation during the transition to independent living. The Resiliency Model assumes that all families face stressors and hardships as part of family life over the life cycle (Figure 1). While some stressors and hardships only require families to adjust (i.e., making small changes in family life), others may constitute a crisis that requires *adaptation* (i.e., significant, major changes in family life to restore order, harmony, and balance within the family system) (27). The transition to independent living in families of AYAs with CP can be understood as requiring *family adaptation*, with significant changes in family living arrangements, roles, responsibilities, emotional and meaning-making processes, and interactions with service systems (6).

During adaptation, families develop competencies, patterns of functioning, and capabilities. They draw from and contribute to networks of supports and resources (27). The model outlines five resiliency factors which shape family adaptation: *family appraisal, family resources, family problem-solving, family coping*, and *patterns of family functioning*. Furthermore, adaptation is affected by a *pile up of demands* on the family system: existing, ongoing, or unexpected stressors, changes, difficulties, and vulnerabilities (27). An examination of these factors enables an in-depth understanding of *how* families successfully respond to stress and hardship, the *contextual and systemic factors* that impact family adaptation, and *the role of policy and practice* in supporting successful adaptation (27, 28).

This two-stage exploratory study aimed to understand the family adaptation process related to the transition to independent living for families of AYAs with CP. It focuses on the process of adaptation, rather than its outcomes (e.g., bonadaptation and positive outcomes, or maladaptation and negative outcomes). Stage 1 of the study [reported elsewhere (6)] used a survey method with family members of AYAs with CP. It provided insight into family members perspectives on the meaning of independent living for the AYA with CP in their family: the opportunity for AYAs to experience adult life; freedom for all parties; uncertainty and worry about safely transferring care; and future planning for ageing family members. Additionally, the first stage also identified some of the barriers and enablers for the transition to independent living: the AYA’s health and wellbeing; the family’s proximity to the AYA; navigating systems; access to funding and equipment; finding suitable accommodation; and confidence in the quality of care (6). Stage 2 of the study provides an in-depth qualitative insight into these factors and the processes that shape family adaptation to the transition to independent living, with implications for policy and practice which align with family perspectives, needs, and experiences.

## 2 Materials and methods

### 2.1 Study design

The overall study used an exploratory, sequential mixed-methods research design, using a survey method followed by interviews informed by the survey findings. This paper reports on Stage 2 of the study, which focused on the factors within the family system that shape families’ resilience and ability to adapt to the AYAs’ transition to independent living. An in-depth qualitative inquiry in the second stage enhanced and deepened the understandings gained from Stage 1 (6). Ethical approval was received from The Royal Children’s Hospital Human Research Ethics Committee (Research Governance) (HREC/89714/RCHM-2022) and St. Vincent’s Hospital Human Research Ethics Committee (HREC #029/23 & SSA ERM/89714).

### 2.2 Participants

Eligible participants were adult (18+ years) family members of AYAs aged 15–30 years diagnosed with CP and receiving treatment from the Transition Support Service (TSS) at The Royal Children’s Hospital and/or at the Young Adult Complex Disability Services (YACDS) clinic at St Vincent’s Hospital in Melbourne, Australia. The TSS assists young people with chronic medical conditions and/or disabilities and their parents/carers to transition to adult care. The TSS partners with adult services such as YACDS to transition healthcare of AYAs with CP. The YACDS clinic provides a multi-disciplinary, statewide service for young people aged 18–40 years with complex disabilities, predominantly CP. Site investigators confer, when needed, with health clinicians to confirm eligibility.

Participants were ineligible if there were known pre-existing risk issues such as significant family conflict, family violence, and housing insecurity that may have been exacerbated by participating in this study. Family members who required an interpreter were ineligible due to a lack of funding to support their participation.

### 2.3 Recruitment

Family members were recruited from the two health services from May 2023 until September 2023. The two site investigators (EC & VS) screened patient records via their clinical and electronic medical record databases. As some AYAs were attending both clinics, site investigators decided who would approach eligible participants about the study to avoid duplication. Following screening, a list of eligible participants and their email contact details was collated at each site (TSS *n* = 61; YACDS *n* = 128), and emails were sent to eligible participants with study information and a link to the online survey with embedded participant information and consent details. An initial email in May 2023, followed by three rounds of reminder emails (June–September 2023) were sent to family members inviting participation. In addition, a printed study information flyer with a QR link to the survey was handed out by the site investigators and health clinicians to eligible participants at both clinics. At the time of consent and completion of the survey as part of the first stage, participants could opt into the second stage to take part in an interview or focus group. The interviews were conducted from November 2023 to March 2024 inclusive.

### 2.4 Interview guide

The findings from the first stage of the study ‘Transition to Independent Living’ (6) informed the second stage, prompting further in-depth discussion with family members about the family adaptation process involved in the transition to independent living and what services can do to support families of AYAs. The Resiliency Model (27) was used to frame open-ended interview questions to explore this adaptation process. The interviewer (MB) is an experienced social work practitioner, educator and research who was not previously known to the participants. Social work professional knowledge, skills and values informed the interview approach and interpretation of the data. To address potential bias, supervision (LCH) was provided during data collection, and transcripts analysed by other members of the research team (HTD, LCH and LKH) with the interviewer confirming interpretations (Supplementary File 1).

### 2.5 Data collection

The interviews were conducted online via Zoom and recorded with the transcription tool. A member of the research team (MB) conducted all the interviews. To ensure confidentiality, the site investigators (EC & VS) did not receive any information regarding family members’ decisions to participate or not. To preserve confidentiality and reduce the risk of identification of participants during collection, analysis and storage of data and information, the following steps were undertaken: participant identifiers were stored separately; the Coordinating Principal Investigator (LCH) was responsible for the storage of a master-file of names and other identifiable data with the participant ID; access was restricted to the study team. There was a separation of the roles of team members responsible for management of identifiers and those responsible for analysing content. The data was analysed by the research team members, who were provided with anonymised data identified only by the unique participant study ID.

### 2.6 Data analysis

Qualitative content analysis, informed by Kuckartz et al. (29) was conducted using the following steps: read and explore data; develop categories; code data; analyse coded data; and present results. Analysis was deductive and theory-based; categories and their definitions were initially developed based on the Resiliency Model (27). Categories were agreed upon by multiple researchers (LCH, HTDN, MB, LKH). Coding occurred in multiple cycles. During this iterative coding process, no new categories needed to be developed, and no significant changes were made to the categories, though the definitions of categories were further developed and refined, ensuring that the category structure captured the complexities present within the dataset (17). A single text passage can be assigned to multiple categories, though refined category descriptions ensured sufficient differentiation between categories (17). Throughout the analytic process, categories and codes were reviewed by multiple researchers (HTDN, MB, LKH, LCH), with disagreements resolved collaboratively. Finally, categories and their codes were summarised and presented in such a way that directly addressed the study’s research question.

Overall, a number of strategies were adopted to enhance the trustworthiness and rigor of this qualitative study (30). Semi-structured interviews allowed for prolonged engagement between the researcher and participants, enabling rapport and an in-depth understanding of participant perspectives. Thorough descriptions of the research context, sampling strategies, and methodological processes helped ensure transparency, allowing others to assess the dependability and transferability of the findings. Reflexive peer debriefing and collaborative analyses addressed confirmability and minimised personal biases through the introduction of alternative perspectives (30).

## 3 Results

Findings from the qualitative content analysis are presented below. Quotes from participants are provided in quotation marks and the participant number noted (e.g., P1). The frequencies of codes assigned to each category are not included in the presentation of results. Frequencies are not useful as the semi-structured interview was highly open-ended, and explicit questions were not asked about every aspect of the Resiliency Model. Therefore, conclusions about significance and relevance based on frequencies should be avoided (17). Quantifying language is used to give some indication of the proportion of the sample who provided relevant responses - ‘most’ indicating 9 to 11 participants; ‘some’ 4 to 8 participants; and ‘few’/‘a small number’ 2 to 3 participants.

### 3.1 Characteristics of participants

The sample included 13 participants (11 individuals and one dyad), representing 12 families. Nine participants identified as mothers, and three identified as fathers of an AYA who lived with CP or a similar neurodevelopmental condition. Ten were partnered or married, and three were unpartnered. The AYAs were aged between 16 and 29, with a mean age of approximately 20.9 years. Nine AYAs were identified by participants as male, and three as female. One AYA was deceased. All AYAs were enrolled with the NDIS.

### 3.2 Vulnerabilities and the pile-up of demands

Alongside the transition to independent living, families experienced multiple stressors, changes, and demands within family life. These vulnerabilities and a pile-up of demands, as defined in the Resiliency Model (27), influence family adaptation. These included: normative transitions (i.e., experiences of growth and ageing that occurred in the same time frame as the transition); relationship strains which resulted from earlier or ongoing care-related stressors (e.g., parents unable to spend time together as a couple); unexpected changes and difficulties internal and external to the family (e.g., programs suddenly becoming unavailable); consequences and difficulties which resulted from family efforts to cope (e.g., mental health difficulties); ambiguity surrounding the transition to independent living (e.g., due to fragmented sources of information); and new patterns of functioning instituted by the family which brought about additional demands or stress (e.g., long commute times to visit the AYA in new accommodation). This accumulation of demands, changes, and strains evolved over time and across life stages, demonstrating that families seldom dealt with a single stressor.

In particular, [the simultaneous] transitions out of the school and children’s health systems were framed as significant family stressors. These transitions involved complex, confusing, and co-occurring processes across multiple systems. The AYA lost the high level of social interactions and activities that they were used to, prompting concerns about boredom or social isolation. Parents were once again responsible for day-to-day care, something they did not expect at their life stage:

*“18-year run, and you have the expectation that at the end of those 18 years you get to step back, maybe a little bit. And instead, we were saying that we were gonna need to step forward and get more involved as school finished.”* (P3)

Participants needed to find services to fill the gaps created by the transitions, such as day programs or in-home support workers. However, options were limited, particularly when the AYA had complex needs. Significant family adaptation needed to occur when options for support were inadequate (e.g., the family moving houses); and in some cases, families were left with no support for these key life transitions.

### 3.3 Family resiliency factors

Shaping family adaptation during the transition were resiliency factors (27) including family appraisal processes; family problem-solving; family coping; and patterns of family functioning. An analysis of these factors highlights the family’s strengths, skills, and resources; and where systems and structures hindered or enabled family adaptation.

#### 3.3.1 Family appraisal processes

In line with the Resiliency model (27), participants appraised the transition to independent living at five levels: *stressor appraisal* (e.g., the transition to independent living as a sustainability issue, with the family needing to *“get it right”* (P1) to avoid regrets and setbacks); *situational* (e.g., aspects of the transition exceeding or not exceeding the family’s capabilities); *family paradigm* (e.g., siblings should have the same choices for independence); *family coherence* (e.g., life has to move on); and *family schema* (e.g., all adults, regardless of disability, need to be independent). Multi-levels of appraisal were part of the family’s efforts to make sense of and manage the stressors of transitioning to independent living (27). Responses demonstrated how appraisal processes shaped confidence about and approaches to the transition. For example, where participants perceived the AYA’s increased confidence, independence, and autonomy over time, this new information was integrated during reappraisal processes, contributing to the parents’ increased sense of confidence about independent living:

*“But as he's got older we realize that he actually does like his independence in his own capacity. And you know, even just a couple of weeks of being at [respite]. They do have a respite programme there, and he's come home, and you know and stated that he would like to be able to attend that with some of his friends that he's met there already, and so my husband and I have thought gosh! Well, we thought that we were doing the best thing for him by having him supported and living at home. But in the future that may not be something that we look at. We, I think independent living is something that he would really enjoy being around.”* (P17)

In contrast, some beliefs shaped participants’ doubts about the transition. For example, a small number of participants articulated that it was a parent’s job to put the AYA first and care for the AYA, thus the transition felt like they were “*abandoning*” (P3, P23) their child, or that they were not a “*loving*,” “*proper*” (P23) parent. This highlighted how appraisal at the family paradigm and family schema level shaped concerns and uncertainty about independent living. One participant provided insight into the external forces that shaped these internal appraisal processes:

*“I think the thing is you get a ridiculous amount of projection on you that you’re kind of an incredibly good person and a saintly person and so, then, if you said, not that I've I don't feel I've bought into that at all. But if you're then suddenly putting your child into…you know, what does that mean about your kind of your love, you're loving, you know, as a mother…”* (P23)

Cultural or social constructions of parenting a child with disability influenced family paradigm and schema, and therefore, the transition to independent living.

#### 3.3.2 Family resources

Families in this study drew on a diverse range of resources to support family life and adapt to the transition to independent living, including *personal*, *family*, and *social resources* (i.e., informal and formal supports) (27). In discussing personal resources, participants reflected on their own and their family members’ personal traits and characteristics [e.g., being “*strong*” (P41), “*persistent*” (P42a)]; organisational, management, and research skills (i.e., which parents had gained throughout their careers, and the AYA’s skills to manage their support workers); parents’ medical knowledge and skills (i.e., from working as a nurse); advocacy skills at the system and policy level; and activities which supported family members’ personal energy to respond to family demands (e.g., hobbies, self-care, emotional coping strategies).

Participants reflected on resources at the family systems level. Family experience and knowledge, energy and persistence, and *“resilience”* (i.e., not being *“disheartened”* by *“roadblocks”* (P17), addressing problems as they arise), supported families in navigating highly complex systems. Patterns of family communication and shared decision-making assisted the transition to independent living; being able to make decisions as a family with a *“unified approach”* (P3) and communicate persistently and openly about the need for independence. Family assets were also beneficial in the transition, expanding the options for independent living (e.g., building a suitable home for the AYA); however, it was noted the purchase of additional properties could create significant financial stress in the family. Overall, personal and family resources enabled families to induce changes and transitions in family life while promoting harmony (27).

Participants also drew on or suggested external social resources that would be helpful for family adaptation. Sources of formal support included multi-media resources (e.g., videos); health and allied health professionals; disability support workers; case managers and support coordinators; disability accommodation; respite programs; day programs; employment, housing, and transport support; schools; and the NDIS. Participants discussed the need for services and advisory bodies specialising in the transition to adulthood and independent living; and *“trial”* programs (P1) for independent living. Sources of informal support accessed or suggested by participants included extended family support or peer support, including from families who had been through the transition. The types and nature of informal and formal supports varied, including but not limited to emotional support; informational support, advice, and guidance; health, mental health, and allied health care; practical support with everyday life and the transition; and disability funding. Supports across mainstream and disability systems were not only for the AYA, but also for family members, throughout the transition and the course of family life.

Responses highlighted where services were or would be particularly helpful. Positive, supportive, and caring relationships with health and disability professionals and support workers were important for the transition and for family life. Participants provided examples of where professionals or support workers were understanding of and responsive to the family’s unique needs and goals; valued the family’s input and expertise in the AYA’s care; took leadership in the provision of care; and advocated for the AYA and the family. Services were also particularly helpful where there was continuity of care and consistency of advice; professionals had extensive knowledge about systems and the services available; there was an appropriate level of support (e.g., one-on-one) for the AYA’s complex needs; and there was flexibility for ongoing family involvement and interaction.

Expert navigation and coordination support for the transition to independent living was identified as particularly important. It was suggested this support can be provided by the hospital team or in the community, with navigators informing families of the opportunities and supports available to the AYA, explaining specific processes, and assisting them with coordination. This support was seen to be crucial. Participants reported significant uncertainty around the transition to independent living, particularly housing and support options; information was not easily accessible, was insufficient, or was fragmented. Navigation support would be an important enabler for the families to navigate this uncertainty and make informed decisions about the transition to independent living:

*“It'd be just wonderful to know. Is there someone out there that could help us navigate?... And at the end of the day it's your choice as a family and [AYA]'s choice as to whether you think that you know this is, he'd be better placed here or here, or here.”* (P17)

This support is necessary, not because families lacked the skills for navigating systems, but, as one participant noted:

*“I prefer to use an agency rather than manage that myself, because they get to do all the interviewing and the compliance and the management. And while I have the life skills and the technical capability of doing that, based on my work in [profession]. I just don't want to. That's a burden and a load I don't need to carry, and it also, I think, keeps me indispensable if I'm managing all of that, and am suddenly removed from the equation.”* (P3)

Participants examined the long-term sustainability of their involvement, and the pile-up of demands during the adaptation process, when considering the need for navigation and coordination support.

#### 3.3.3 Family problem-solving

Participants demonstrated how families utilised strategies to *organise the transition into manageable components; identify courses of action to address each component; and initiate steps to address the components* (27). Families devoted time to researching support and independent living options for the AYA, visiting online information, engaging with professionals and peers, visiting services, and trialling different programs and environments. They broke the task down into smaller components, steps that needed to be undertaken; and where possible, undertook preparatory work (e.g., will planning) and involved other people (e.g., support workers, health professionals) in the process. Participants identified the need to have a pragmatic approach, to communicate, to utilise checklists, and to deal with one problem at a time as they arise. In thinking through different aspects of independent living, they anticipated needs or problems that might occur, particularly in relation to the AYA’s care, health, and safety. Plans and multiple back-up plans were created, addressing anticipated problems to a high level of specificity:

*“So I think it's just being aware and not going into it within the unknown. So really thinking about all the different scenarios. Okay, how? How are you gonna get from, you know, transport to the front door and you know what you know. Transport, what? How are we gonna get, how you gonna get around? And just yeah, trying to think of all the different scenarios. I mean, you can't cater for everything. And sometimes in the community, you know, you're gonna come up against something. But just yeah, as best as you can, try and prepare for, yeah, what about for you personally preparing for day to be living.”* (P25)

Measures were put in place in anticipation of challenges or changes, particularly around capacity building. Helping the AYA build independent living skills, training support staff, or writing down information about the AYA’s care ensured that parents were not the only “*holders of all this knowledge”* (P23). Some families set short-term goals for gradually increased independence. Where there were significant structural barriers, a small number of participants engaged in advocacy, (e.g., with the NDIS, with politicians, or in the community). Therefore, participants called on a range of problem-solving skills and strategies to manage, reduce, or eliminate stressors and facilitate adaption to independent living transitions (27).

For some families, starting the transition early was an important part of family problem-solving, though they had varied experiences and perspectives. A small number of participants described having started long-term planning when the AYA was in early childhood. Some others started the process when the AYA was moving into the young adult age group, out of the school system, or out of children’s services; and/or when the parents moved towards retirement. A few participants expressed their hesitation with starting the process when the AYA reached young adulthood, acknowledging mixed feelings and worries about the AYA. For a couple of participants, the process did not start until parents received diagnoses of serious health conditions. The benefits of starting early were discussed by some participants: transition is a long process dependent on NDIS funding, and early planning allowed families to explore options, take small steps over time, transition slowly and smoothly, get used to the idea of independence, build the right support team, and emotionally process the transition. It was also suggested that *“it’s never too early to think about it,”* “*the sooner people start the better”* (P25); and that schools and health professionals can start the conversation, provide information, and link families with services as early as 14 to 16 years of age.

#### 3.3.4 Family coping

Families coped with the transition by *eliminating or reducing demands*; *managing tensions and family strains*; *creating, shaping, and evaluating meaning*; and *acquiring additional resources* (27). To reduce the intensity of the demands associated with the transition, families planned steps to practise independence, with the AYA being away from their parents for increasing amounts of time; at home, in day programs/activities, in a separate family home (with or without support workers), or in respite. This was for the benefit of both the AYA and their parents. These ‘practice’ periods allowed parents to monitor the AYA’s health and wellbeing, in line with the family’s goals for the AYA:

*“I like when she goes, it is often a bit more short-term, and so I get to see her afterwards, like she'd been in for 4 nights, and she came home. So, her pressure area on her toes became water blisters. So, you know, things like that. And they're still from 12 months ago in hospital. So, it just you at least get to monitor her physically as well, or you know, her PEG site is really red and inflamed and things like that.”* (P5)

Small, experimental steps allowed the AYA and their parents to gain confidence in the transition, build familiarity with separation, and prevent sudden, traumatic changes:

*“You just can't have her living at home, and then push comes to shove. And then overnight she's in a strange place crisis, and in crisis and traumatised by the change and all that, we're trying to make the transition to independent living is least traumatic for her as possible.”* (P8)

At times, participants found ways to take a break, from caring responsibilities (e.g., by accessing respite) or from the demands of the transition (e.g., pausing the process and re-starting later). Taking individual time out, or spending time together as a couple or family, assisted with the management of family tensions resulting from ongoing care-related stressors. Coping was practical, but also emotional; participants engaged in emotional and appraisal processes to make the transition more manageable or acceptable. For example, participants examined and accepted the fear, grief, and complicated feelings that came with an adult child leaving the family home.

Families acquired additional resources, including both formal and informal supports described in previous sections, to cope with the transition to independent living. However, systemic issues impacted the family’s acquisition of social resources, including issues with the NDIS and the NDIS service providers. Participants discussed the delays and slowness of NDIS processes; limited funding for a high-level of care and the right care ratio; caps on respite use; and the lack of options for respite and accommodation facilities in the current one-size-fits-all system. They were concerned about the quality of services, including respite facilities and Specialist Disability Accommodation (SDAs), which were not as advertised (e.g., falsely indicating their capacity to respond to ‘high and complex needs’); and the lack of transparent information about behaviours of concerns amongst other service users of disability programs and facilities, which could put the AYA at risk. Furthermore, participants reported that the NDIS discouraged the use of respite and support coordination, despite the importance of these services to family coping and adaptation.

Notwithstanding families’ efforts and skills at acquiring support worker to assist with family coping, this remained a long, intensive, and unpredictable process shaped by systemic issues. Participants highlighted the challenges with finding support workers who were appropriately trained and competent in responding to complex needs, particularly medical needs (e.g., PEG feeding and insulin injections). One participant suggested this was due to the siloed nature of the disability and health systems:

*“And then they need to be able to train up staff to, you know, and there's this thing between the NDIS and the medical that they don't do medical stuff, but in his case it's all part of his disability.“* (P23)

A few others highlighted workforce retention issues, with families needing to “*start all over again”* (P41) when losing suitable, competent workers. One participant reported service providers’ requirements to provide longer shifts for individual workers, but the lack of NDIS funding to do so, which put the family at risk of losing access to supports.

In acquiring additional resources for the transition, families engaged with extended family to different extents. Some participants positioned extended family as a safety net and support network for the AYA in the long-term:

*“I have absolute confidence that if (husband) and I got hit by bus, somebody in the family would step up, and they would see themselves in the same unthinkable situation that we were in, that somebody needs to look after him. I know they do it differently. and how we do it. and that's fine. But they would look after him.”* (P3)

In contrast, some participants felt they were not able to acquire support from extended family. They felt it was too much to continue asking for extended family to participate in the AYA’s care as it was “*a bit over and above any normal family sort of thing*” (P23); extended family lived too far away; or family members died, leaving “*a gap in [their] family connection”* (P10). One participant felt extended family did not understand their family’s day-to-day reality; and as they became overly familiar with the AYA’s ongoing issues, they became less likely to check in and provide emotional support. This highlighted changes in family resources over time.

#### 3.3.5 Patterns of family functioning

Finally, the families’ patterns of functioning facilitated their adaptation during the transition to independent living (27) McC. These include patterns of functioning within the family and those in relation to the community (i.e., the family’s interactions with formal supports). For example, for most participants, it was important that family was involved in the transition and retained their close involvement in the AYA’s life, highlighting the need for regular and flexible opportunities to spend time with and provide care to the AYA. Some participants spoke of needing to institute patterns of functioning whereby family boundaries were extended, and caring knowledge and responsibilities shared with professionals and support workers. It was identified that regular communication and feedback was crucial in the initial stages of accessing a service, allowing parents to train support workers in caring for the AYA; to understand how the AYA was being cared for and trouble-shoot as necessary; and overall, to build trust in the supports provided:

*“We just trusted [support workers] so much part of our family. He was part of their family. That would have been maybe an option. Trust is earned and not you know, expected. So, they really well and truly had our back, and we really miss them.”* (P42a)

Some participants believed this could eventually lead to a new pattern of functioning whereby professionals and support workers were involved in decision-making or given decision-making capacity over the AYA’s care.

There were few references to family’s rules and boundaries; coalitions in the family; and patterns of communication—which are components of the Resiliency Model (27). It is noted that participants were not explicitly asked to consider patterns of family functioning.

## 4 Discussion

This exploratory study examined the family adaptation process during the transition to independent living for families of AYAs with CP (27). The context for this study is Australia, where the NDIS began to be implemented progressively across Australia from 2013, with full implementation completed by 2018. The families in this study have experienced the NDIS firsthand as the AYAs are aged between 16 and 29 years and enrolled in the scheme. Given the transformative nature of the NDIS, this study is timely in critically examining the current and evolving landscape and its potential to affect the life trajectories among young people with CP and their families as they embark on the transition to independent living.

Overall, findings highlight the factors and resources, both internal and external to the family, which shaped participants and their families’ experiences of adaptation. The Family Resiliency Model’s focus on family appraisal processes, family problem-solving, family coping and patterns of family functioning helped identify specific family processes that practitioners can be more aware of. For example, this study found parents’ appraisals about independent living positively changed when AYA’s felt confident and autonomous; and that parents were resourceful and active in researching independent living options. Families in this study brought these strengths, resources, skills, and strategies to a transition process that is often met by rigid policies, unfamiliar and complex bureaucratic systems, market-driven services, and disability workforce issues. These structural barriers have been highlighted in previous studies of young people with disability during their transition to adulthood (6, 15, 20, 25). Our study’s unique contribution is an in-depth description of how family adaptation occurs in response to structural barriers, to ensure a safe, gradual, and sustainable transition to independent living amongst the AYAs with CP in our sample.

Findings are consistent with the research evidence, highlighting the importance of transition services which support AYAs and their families to navigate complex systems, bureaucratic requirements, and fragmented information sources (1, 6, 18). Navigation needs identified by family members in this study does not relate only to the disability system for independent living solutions, but also to the education and healthcare systems, as the transitions out of school and paediatric healthcare occur around the same time as the transition to independent living. However, the NDIS Review (17) did not examine or recognise the co-occurrence of these transitions, and the ‘pile-up of demands’ this places on families.

Our findings highlight that all health and disability services, including transition services, need to demonstrate family systems thinking without focusing solely on the individual with disability. This family systems thinking is essential, considering the centrality of family adaptation to successful transitions across systems and into adulthood. Health and disability policies and services need to recognise family resiliency factors including their knowledge, research, anticipatory thinking, and problem-solving strategies for system and service navigation. Family members in this sample also held extensive knowledge about the AYAs’ care needs and strategies for safe, effective care. Family expertise needs to be incorporated in service delivery, creating stronger relationships between the family system and the support system. As demonstrated in this study, collaborative relationships between the family and service providers, whereby family members’ input, involvement, and expertise are valued and promoted, positively contributed to the independent living transition and to family life more broadly.

This study found that participants navigated multiple systems to support not only the AYA’s, but their own health and wellbeing as parents/carers during the transition process. The National Carer Strategy (26) highlighted that carers with chronic health issues “need to access multiple complex government systems to coordinate supports both for themselves and for the person they care for,” pointing to additional demands impacting family adaptation. Several of the participants in this sample had chronic health issues which they navigated around the same time as the AYA’s transition to adulthood. This has implications for policy and practice which recognises the needs of families with ageing parents.

The Resiliency Model (27) provided a useful framework for examining the family system in context, demonstrating how families make adaptations to meet the changing needs of the AYA and of family members. The model was particularly useful in highlighting the strengths, skills, and resources internal to the family, enabling for a sustained focus on resilience rather than deficits. It was also helpful in elucidating the ‘pile-up’ of demands that families experienced over time, promoting a holistic and comprehensive understanding of the family system above and beyond the stressors of independent living transitions. It is recommended that future research examines how the model may be enhanced to understand and support family adaptation during the transition to independent living, with implications for both policy and practice.

There are some limitations to this study. Stage 2 of the study included a small sample from one Australian state. Data saturation was not reached in this exploratory study with convenience sampling within a small population. However, the use of a small sample and semi-structured interviews allowed for a highly in-depth examination of family adaptation processes within context. The sample primarily consisted of women; however, this likely reflects broader gender trends in family caregiving in Australia, i.e., women over-represented as primary carers (26). Due to funding limitations, individuals who required an interpreter were not invited to participate in the study. Due to ethical considerations, the study also excluded individuals experiencing psychosocial risks that would be exacerbated by participation in research, given our focus on family processes. Considering these characteristics of the sample, and that structural contexts (e.g., services and systems) vary across Australia and internationally, there is no assumption about the generalisability of this study’s findings. Future studies should seek data saturation with a larger and more representative sample. Further research should also explore potential differences in the experiences of culturally and linguistically diverse families, and families experiencing significant psychosocial risks. Member checking may enhance the trustworthiness of findings, providing participants with opportunities to validate or expand on interpretations (30).

This study on parents’ perspectives provides valuable insights which will be complemented by future studies on AYAs’, siblings’, and other family members’ perspectives and experiences of the transition to independent living. This exploratory, cross-sectional study was also unable to track the transition, family adaptation, and its outcomes over time (e.g., bonadaptation or maladaptation); this is an important area for further examination in future research.

## 5 Conclusion

The transition to independent living is a highly complex process which requires families to navigate multiple bureaucratic systems, ambiguous processes, and at times, inadequate services. Such systemic issues have been recognised in policy, practice, and research, but they remain. This study details and highlights how families draw on family strengths and resources to appraise, problem-solve, and cope with systemic barriers, as well as the many practical and emotional demands associated with independent living transitions for AYAs with CP. This study offers a strengths-based and systems-informed understanding of family processes and family resiliency, which is directly relevant to both policy and practice. System navigation and family-centred support can promote the factors that assist with family adaptation, enabling families to lead a transition process that is safe, gradual, and sustainable.

## Data Availability

The datasets presented in this article are not readily available because the participants of this study did not give written consent for their data to be shared publicly, so due to the sensitive nature of the research supporting data is not available. Requests to access the datasets should be directed to Lyndal Hickey, hickeyl@unimelb.edu.au.
