# Supplementary material for: Independent living transitions for young people with cerebral palsy in Australia: aligning policy and practice with family realities
Source: Front Public Health. 2026 Feb 19;14:1755553. doi: 10.3389/fpubh.2026.1755553 (PMC12960535; doi:10.3389/fpubh.2026.1755553)
Supplement: Supplementary file 1 [file Table_1.docx]

**Supplementary File 1: Transition to Independent Living – Family Member Focus Group and Interview Guide**

1. **[Key area: Family resiliency factors that influence the transition to independent living]**

The survey responses have revealed [add in themes and what makes it harder or easier to transition to independent living].

1. Meaning the family member attributes to the situation.

Reflecting on your experience in your family:

- What is your response to what we have learned so far from families about the transition to independent living?
- How are your views about the meaning of the transition to independent living the same or different?

Reflecting on the broader experience of families:

- What shapes the meaning families give to the transition to independent living?
- When do families begin to think about the transition to independent living?

1. Family resources

Reflecting on the broader experience of families:

- What family resources are needed to support the transition to independent living? [Personal resources, Family system resources, Social supports - friends, services]
- How can family resources be used to support the transition to independent living?
- When are family resources needed to support the transition to independent living?

1. Family coping processes

Reflecting on the broader experience of families:

- What coping strategies could be used within families to support the transition to independent living? [Positive coping strategies, Negative coping strategies]
- How do families use coping strategies to support the transition to independent living?
- When do families need to use coping strategies to support the transition to independent living?

1. Problem-solving abilities

Reflecting on the broader experience of families:

- What problem-solving strategies could be used within families to support the transition to independent living?
- How do families use problem-solving strategies to support the transition to independent living?
- When do families need to use problem-solving strategies to support the transition to independent living?

1. **[Key Area: Supporting families of young people with CP to transition to independent living]**

- What services could support the transition to independent living? [For families of young people with CP, For young people with CP]
- What types of support are needed and to whom?
- When do you think these services could provide this support?
- To what extent do you think where you live impacts on availability or access to services to support the transition to independent living?

1. **Is there another thing you would to share with other families or had wished you had done differently? Anything else you would like to share with families about the transition to independent living?**
